# Acidosis Drives Damage-associated Molecular Pattern (DAMP)-induced Interleukin-1 Secretion via a Caspase-1-independent Pathway*

**DOI:** 10.1074/jbc.M113.478941

**Published:** 2013-09-10

**Authors:** Michelle E. Edye, Gloria Lopez-Castejon, Stuart M. Allan, David Brough

**Affiliations:** From the Faculty of Life Sciences, University of Manchester, Manchester M13 9PT, United Kingdom

**Keywords:** Acidosis, Caspase, Glia, Inflammation, Innate Immunity, Interleukin, Stroke

## Abstract

The proinflammatory cytokine IL-1β is a key mediator of inflammatory responses that contribute to and exacerbate brain injury. IL-1β is synthesized by microglia in the brain as an inactive precursor (pro-IL-1β). Cleavage of pro-IL-1β to a mature form is stimulated by damage-associated molecular patterns (DAMPs). These DAMPs are sensed by a pattern recognition receptor called NLRP3, which forms an inflammasome, resulting in the activation of caspase-1 and cleavage of pro-IL-1β. To date, regulation of the inflammasome in culture has been studied under normal culture conditions, and it is not known how DAMPs signal under disease relevant conditions such as acidosis. Given the presence of acidosis in pathological states, our objective was to test the hypothesis that acidic conditions modify DAMP-induced IL-1β release from cultured primary mouse glial cells. When LPS-primed glial cells were stimulated with DAMPs under acidic conditions (pH 6.2), the predominant IL-1β form secreted was the 20-kDa rather than the 17-kDa caspase-1-dependent species. Lactic acidosis, induced by the addition of 25 mm lactic acid, also induced the release of 20-kDa IL-1β. This 20-kDa product was produced independently of NLRP3 and caspase-1 but was inhibited by the cathepsin D inhibitor pepstatin A. These data suggest that under disease relevant acidosis, DAMPs and lactic acid induce the secretion of IL-1β independently of the inflammasome. Therapeutic strategies directed to the inhibition of IL-1β processing should therefore consider alternative processing of IL-1β in addition to caspase-1-dependent processing.

## Teaser

**Background:** IL-1β is a “master” proinflammatory cytokine central to host responses to injury and infection. Danger molecules (DAMPs) activate IL-1β via activation of a protease called caspase-1.

**Results:** Acidosis promotes alternative DAMP-induced processing of IL-1β independent of caspase-1.

**Conclusion:** Acidosis is a regulator of inflammatory pathways.

**Significance:** Multiple pathways may contribute to the activation of IL-1 during disease.

## Introduction

Non-communicable diseases (cardiovascular disease, diabetes, stroke, and cancer) kill more people than all other causes combined and are thus recognized as a global healthcare priority (1, 2). Inflammation has been strongly implicated in non-communicable diseases (3) and, in this context (the absence of infection), is considered “sterile” and represents a major drug target (4). IL-1β is a primary mediator of sterile inflammatory responses, and given its established contribution to disease (5), understanding the mechanisms of its processing and release could lead to the identification of new therapeutic targets. IL-1β is an established contributor to acute brain injury, such as caused by stroke (6).

In the brain, IL-1β is produced primarily by microglia in response to an inflammatory stimulus as an inactive precursor (pro-IL-1β). Pro-IL-1β requires proteolytic cleavage to an active mature form that signals at IL-1 receptor I on responsive cells (7). Activation of IL-1β is regulated by inflammasomes, which are multimolecular complexes defined by the presence of a cytosolic pattern recognition receptor. Of particular relevance to sterile inflammation is the pattern recognition receptor NLRP3 (NLR family, pyrin domain-containing 3) (8). After activation, NLRP3 interacts with the adaptor protein ASC (apoptosis-associated speck-like protein containing a CARD) and procaspase-1 to form an inflammasome, resulting in activation of caspase-1 and its processing of pro-IL-1β to a mature active form (9). NLRP3 senses molecules released from dead and dying cells, so-called damage-associated molecular patterns (DAMPs)^2^ (10). Treatment of activated macrophages with necrotic cells also drives NLRP3 inflammasome-dependent IL-1β secretion (11). How diverse DAMPs activate NLRP3 is not known, but several host signals are thought to integrate these stimuli (9). To date, studies investigating regulation of the inflammasome *in vitro* have been conducted under physiological or “normal culture” conditions. However, during disease, there are profound changes in the intercellular milieu (such as a drop in pH), and how DAMPs signal under these particular conditions is not known.

Lactic acidosis commonly occurs during disease conditions and results from an increase in anaerobic glycolysis due to tissue hypoxia. In the brain following a stroke, parenchymal pH can drop as low as 6.2 (12). It was reported recently that P2X7 receptor-dependent release of IL-1β from cultured microglia is affected by acidosis (13). Stimulation of microglia with the P2X7 receptor agonist ATP at pH 6.2 results in the secretion of a 20-kDa species of IL-1β as opposed to the more conventionally reported 17-kDa form. The 20-kDa cleavage product does not require caspase-1 but rather depends upon the protease cathepsin D (13). Several other non-caspase proteases also cleave pro-IL-1β (14, 15), and proteinase-3 has been implicated in acute arthritis (16). Acidosis was also recently described as a danger signal that could activate the NLRP3 inflammasome (17). Thus, during disease, pro-IL-1β can be cleaved by a variety of proteases that may act to shape the inflammatory response.

Here, we sought to determine whether acidic conditions modify DAMP-induced IL-1β release from cultured primary glial cells. We report that acidosis induced a shift from the 17-kDa to the 20-kDa IL-1β product in response to DAMP stimulation and that this was independent of the NLRP3 inflammasome. We also report that the addition of lactic acid directly to the culture induced the secretion of 20-kDa IL-1β, raising the possibility that lactic acidosis could drive IL-1-dependent inflammatory responses during disease.

## EXPERIMENTAL PROCEDURES

### 

#### 

##### Materials

RPMI 1640 medium, DMEM, penicillin/streptomycin solution, LPS (*Escherichia coli* O26:B6), ATP, l-lactic acid, cathepsin D inhibitor pepstatin A, and cathepsin B inhibitor CA-074 methyl ester were purchased from Sigma. The caspase-1 inhibitor acetyl-YVAD-aldehyde (Ac-YVAD-CHO) and calpain inhibitor III were purchased from Calbiochem. Monosodium urate (MSU) and calcium pyrophosphate dihydrate (CPPD) were purchased from InvivoGen. FBS was purchased from PAA Laboratories. HEPES-buffered salt solution comprised 145 mm NaCl, 2.5 mm KCl, 1 mm MgCl_2_, 1.8 mm CaCl_2_, 20 mm HEPES, 10 mm glucose, and 0.01% BSA as described previously (13). The pH of the HEPES-buffered salt solution was adjusted using 1 m NaOH. Antibodies against human and mouse IL-1β and mouse IL-1α were purchased from R&D Systems. HRP-conjugated secondary antibodies were from DAKO.

##### Cell Culture

Initial studies used the human monocytic cell line THP-1, which provides a well characterized secretion model of IL-1β. THP-1 cells were cultured in RPMI 1640 medium supplemented with 10% FBS, 100 units/ml penicillin, and 100 μg/ml streptomycin. Cells were plated in 24-well plates at a density of 5 × 10^5^ cells/well and treated with phorbol 12-myristate 13-acetate (0.5 μm). After 3 h, the medium was removed, fresh medium was added, and cells were incubated overnight (37 °C, 5% CO_2_). Cells were then LPS-primed (1 μg/ml, 4 h) to induce pro-IL-1β and NLRP3 expression. The culture medium then replaced with HEPES-buffered salt solution for subsequent treatments.

DAMP-dependent inflammatory responses in cultures of mixed glia reflect inflammatory responses produced by an ischemic brain (18), so we considered these cultures to represent an appropriate system for examining the effects of pH on DAMP-induced inflammation in the brain. Primary mixed glia from 1–4-day-old mice (C57BL/6J or NLRP3 knock-out (KO)) were cultured as described previously (18). Briefly, meninges were removed, and cells were dissociated by trituration. Cells were seeded at one brain/60 cm^2^ and maintained in growth medium (DMEM supplemented with 10% FBS, 100 units/ml penicillin, and 100 μg/ml streptomycin). Cells were incubated at 37 °C in 5% CO_2_ until confluent (∼14 days *in vitro*). Cultures were then incubated with LPS (1 μg/ml, 18–24 h). The growth medium was replaced with serum-free medium or HEPES-buffered salt solution prior to treatment. All procedures were performed in accordance with the Animal (Scientific Procedures) Act 1986.

##### Treatments

Prior to treatment with DAMPs (ATP, MSU, and CPPD), the culture medium was replaced with HEPES-buffered salt solution with the pH adjusted to 7.4 or 6.2. Prior to lactic acid treatment, the culture medium was replaced with serum-free medium. Primed human THP-1 or mouse mixed glial cultures were treated with ATP (5.5 mm, 1 h), MSU (250 μg/ml, 1 h), CPPD (250 μg/ml, 1 h), or lactic acid (25 mm, 8 h). In subsequent studies, cells were pretreated with caspase-1 (Ac-YVAD-CHO, 100 μm), cathepsin B (CA-074 methyl ester; 50 μm), and cathepsin D (pepstatin A, 50 μm) inhibitors or their respective vehicles (1% Me_2_SO or MeOH) for 15 min prior to DAMP/lactic acid treatment. Supernatants were collected and stored at −20 °C until required.

##### Western Blotting

Supernatants were collected and, if required, concentrated using 10-kDa cutoff filters (Millipore). Samples of this supernatant were resolved on 12% polyacrylamide gels for detection of IL-1β or IL-1α. Proteins were transferred to a nitrocellulose membrane, and specific proteins were detected by Western blotting with anti-human IL-1β, anti-mouse IL-1β, or anti-mouse IL-1α, followed by a secondary HRP-conjugated antibody, and subsequently detected using enhanced chemiluminescence reagents (ECL, Amersham Biosciences).

##### ELISA

Supernatants were analyzed for IL-1β and IL-1α using specific ELISA kits for human or mouse IL-1 from R&D Systems according to the manufacturer's instructions.

##### Cell Death Assay

Lactate dehydrogenase (LDH) assay was used as an indicator of cell death. LDH release from cells was detected using the CytoTox 96® non-radioactive cytotoxicity assay (Promega) according to the manufacturer's instructions.

##### Statistics

Statistical analysis was performed using GraphPad Prism v5. For comparisons between two groups, Student's *t* test was used. For comparisons involving three or more experimental groups, a one-way analysis of variance (ANOVA) with a Bonferroni multiple-comparisons post hoc test was used. Data are expressed as means ± S.E. for the number of repeats indicated in the figure legends. *p* ≤ 0.05 was considered significant.

## RESULTS

### 

#### 

##### DAMPs Induce Alternative IL-1β Processing at Acidic pH in THP-1 Cells

**FIGURE 1.**
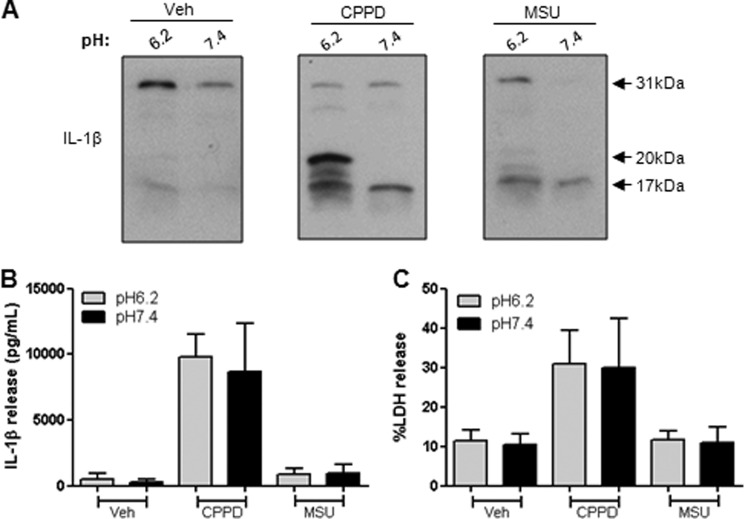
**Effect of extracellular pH on IL-1β processing in THP-1 cells.** LPS-primed THP-1 cells were treated with vehicle (*Veh*), CPPD (250 μg/ml), or MSU (250 μg/ml) for 1 h at pH 6.2 or 7.4. Processing of IL-1β was observed by Western blotting of the supernatants (*A*), with quantification of levels released by ELISA (*B*). The effect of pH and DAMPs on cell death was measured by the release of LDH (*C*). ELISA and LDH data are means ± S.E. of three separate experiments. Western blots are representative of three separate experiments.

In LPS-primed THP-1 cells incubated at pH 6.2, mature IL-1β release was not observed in the absence of DAMP, in contrast to a previous report (17). However, at pH 6.2, both 20- and 17-kDa forms were present after treatment with the NLRP3 inflammasome-activating DAMPs CPPD and MSU (Fig. 1*A*). At pH 7.4, only the 17-kDa form was present in the culture supernatants after DAMP treatment (Fig. 1*A*). In these THP-1 cells, CPPD proved to be a powerful inducer of IL-1β release, with levels readily detected by ELISA (Fig. 1*B*). There was no difference in DAMP-induced toxicity between cells maintained at pH 7.4 and 6.2 (Fig. 1*C*), suggesting that the effect cannot be explained by toxicity of the pH change. Thus, these data indicate that at acidic pH (pH 6.2), DAMPs induce the release of 20-kDa IL-1β from THP-1 cells.

**FIGURE 2.**
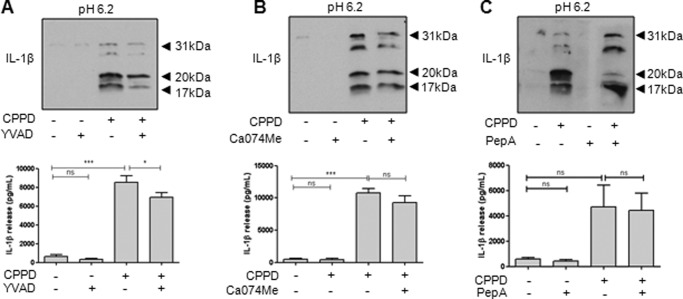
**DAMP-induced release of 20-kDa IL-1β is caspase-1-independent.** LPS-primed THP-1 cells were incubated at pH 6.2 and treated with the caspase-1 inhibitor Ac-YVAD-CHO (*YVAD*; 100 μm; *A*), the cathepsin B inhibitor CA-074 methyl ester (*Ca074Me*; 50 μm; *B*), or the cathepsin D inhibitor pepstatin A (*PepA*; 50 μm; *C*) for 15 min prior to CPPD treatment (250 μg/ml, 1 h). Processing of IL-1β was observed by Western blotting of the supernatants (*blots*), with quantification of levels released by ELISA (*graphs*). ELISA data are means ± S.E. of three separate experiments. Western blots are representative of three separate experiments. Statistical analysis was performed using a one-way ANOVA with a Bonferroni post hoc test. *ns*, not significant; *, *p* < 0.05; ***, *p* < 0.001.

We subsequently showed that neither the caspase-1 inhibitor Ac-YVAD-CHO (Fig. 2*A*) nor the cathepsin B inhibitor CA-074 methyl ester (Fig. 2*B*) blocked the production of 20-kDa IL-1β from LPS-primed THP-1 cells induced by CPPD at pH 6.2, thereby suggesting alternative processing of IL-1β independent of caspase-1 in THP-1 cells at acidic pH. Consistent with the previously reported effect of acidic conditions on P2X7 receptor-dependent IL-1β release (13), the cathepsin D inhibitor pepstatin A inhibited CPPD-induced release of 20-kDa IL-1β from LPS-primed THP-1 cells, whereas the release of 17-kDa IL-1β was unaffected (Fig. 2*C*). Overall, these data suggest that in THP-1 cells at pH 6.2, CPPD induces the release of a cathepsin D-dependent but not caspase-1-dependent IL-1β that is 20 kDa in size.

##### DAMPs Induce Alternative IL-1β Processing at Acidic pH in Mixed Glial Cultures

**FIGURE 3.**
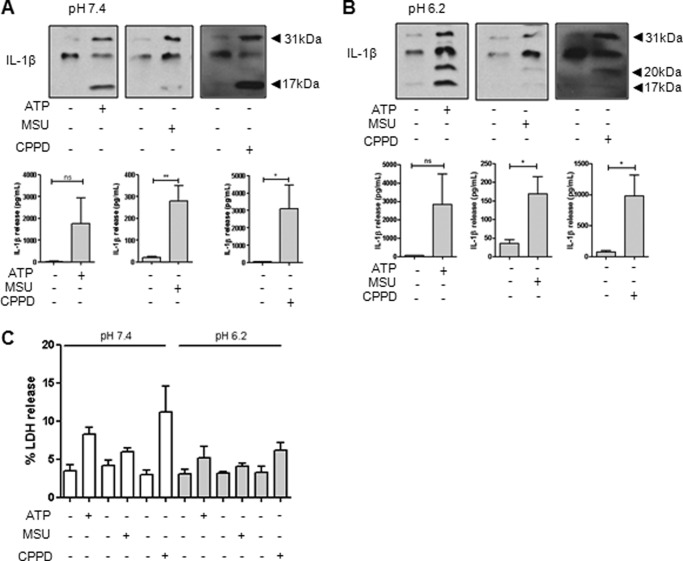
**Effect of extracellular pH on IL-1β processing in primary mixed glia.** LPS-primed mixed glia were treated for 1 h with ATP (5.5 mm), MSU (250 μg/ml), or CPPD (250 μg/ml) at pH 7.4 (*A*) or pH 6.2 (*B*). Processing of IL-1β was observed by Western blotting of the supernatants (*blots*), with quantification of levels released by ELISA (*graphs*). The effect of pH and DAMPs on cell death was measured by the release of LDH (*C*). ELISA and LDH data are means ± S.E. of between five and six separate experiments. Western blots are representative of three separate experiments. Statistical analysis was performed using Student's *t* test. *ns*, not significant; *, *p* < 0.05; **, *p* < 0.01.

We next determined whether the alternative caspase-1-independent processing of IL-1β in response to DAMPs at acidic pH occurs in primary cultures of glia. Incubation of LPS-primed mouse mixed glia at pH 7.4 with the NLRP3 inflammasome-activating DAMPs ATP, MSU, and CPPD induced the secretion of 17-kDa IL-1β as expected (Fig. 3*A*). However, when the cultures were incubated under acidic conditions at pH 6.2, these DAMPs also induced the secretion of 20-kDa IL-1β (Fig. 3*B*), consistent with a previous report on the effects of ATP (13) and our data presented above in the THP-1 cells (Fig. 1). There were no obvious differences in DAMP-induced toxicity between the experiments at different pH values (Fig. 3*C*), suggesting that the effect cannot simply be explained by toxicity of the pH change.

**FIGURE 4.**
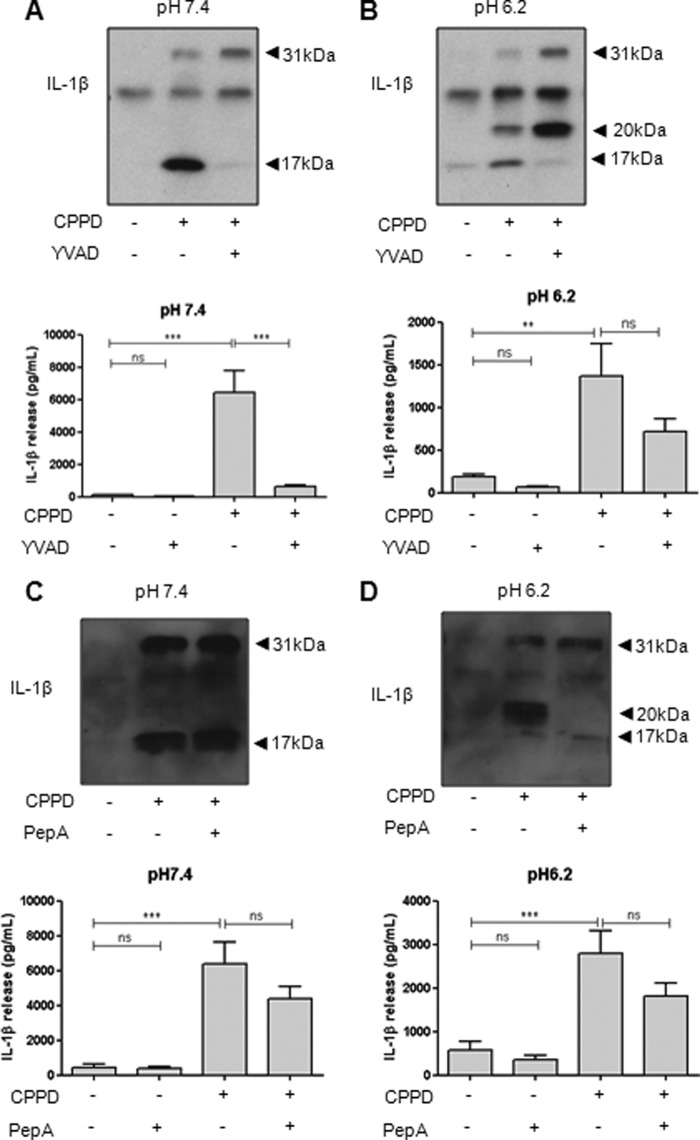
**DAMP-induced release of 20-kDa IL-1β is caspase-1-independent in primary mixed glia.** LPS-primed mixed glia were pretreated with the caspase-1 inhibitor Ac-YVAD-CHO (*YVAD*; 100 μm) or the cathepsin D inhibitor pepstatin A (*PepA*; 50 μm) for 15 min prior to CPPD treatment (250 μg/ml, 1 h) at pH 7.4 (*A* and *C*) or pH 6.2 (*B* and *D*). Processing of IL-1β was observed by Western blotting of the supernatants (*blots*), with quantification of levels released by ELISA (*graphs*). ELISA data are means ± S.E. of between five and six separate experiments. Western blots are representative of three separate experiments. Statistical analysis was performed using a one-way ANOVA with a Bonferroni post hoc test. *ns*, not significant; **, *p* < 0.01; ***, *p* < 0.001.

To investigate 20-kDa IL-1β released from mixed glia is processed in the same way as that released from THP-1 cells, we pretreated cells with the caspase-1 inhibitor Ac-YVAD-CHO. CPPD treatment of LPS-primed mixed glia at normal pH (7.4) resulted in the release of mature 17-kDa IL-1β that was completely inhibited by Ac-YVAD-CHO pretreatment (Fig. 4*A*). At pH 6.2, Ac-YVAD-CHO still inhibited the appearance of 17-kDa IL-1β in response to CPPD but had no effect on 20-kDa IL-1β (Fig. 4*B*). Conversely, pepstatin A had no effect on CPPD-induced IL-1β (17 kDa) secretion at pH 7.4 (Fig. 4*C*), but at pH 6.2, pepstatin A inhibited the release of 20-kDa IL-1β (Fig. 4*D*). This confirmed that production of 20-kDa IL-1β was independent of caspase-1 and suggested that it required cathepsin D.

**FIGURE 5.**
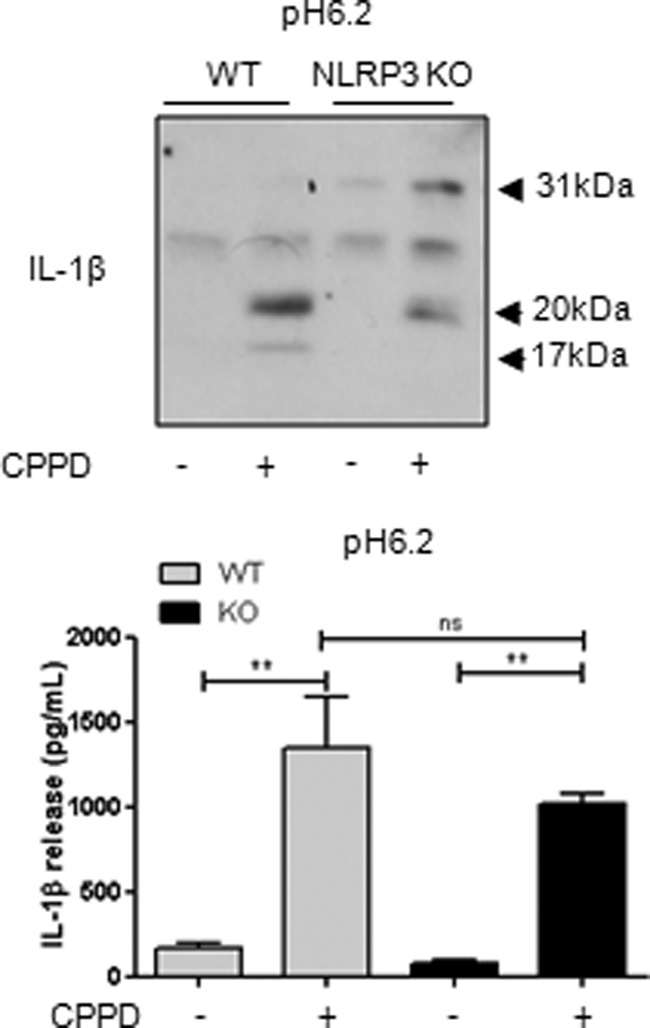
**CPPD-induced 20-kDa IL-1β is NLRP3-independent in primary mixed glia.** Mixed glial cultures prepared from WT and NLRP3 KO mice were treated with CPPD (250 μg/ml, 1 h) at pH 6.2. Processing of IL-1β was observed by Western blotting of the supernatants (*blot*), with quantification of levels released by ELISA (*graph*). ELISA data are means ± S.E. of three separate experiments. Western blots are representative of three separate experiments. Statistical analysis was performed using a one-way ANOVA with a Bonferroni post hoc test. *ns*, not significant; **, *p* < 0.01.

To confirm that the effects of the NLRP3-activating DAMP CPPD at acidic pH were independent of the NLRP3 inflammasome and caspase-1, we compared the effects of CPPD on IL-1β release in cultures of mixed glia isolated from WT and NLRP3 KO mice. At pH 6.2, CPPD induced the secretion of 20-kDa IL-1β from both WT and NLRP3 KO mixed glia (Fig. 5), confirming that NLRP3 inflammasome-activating DAMPs induced the secretion of IL-1β from LPS-primed cells independently of the NLRP3 inflammasome under acidic conditions.

##### Lactic Acidosis Induces IL-1β Release from Mixed Glial Cultures

**FIGURE 6.**
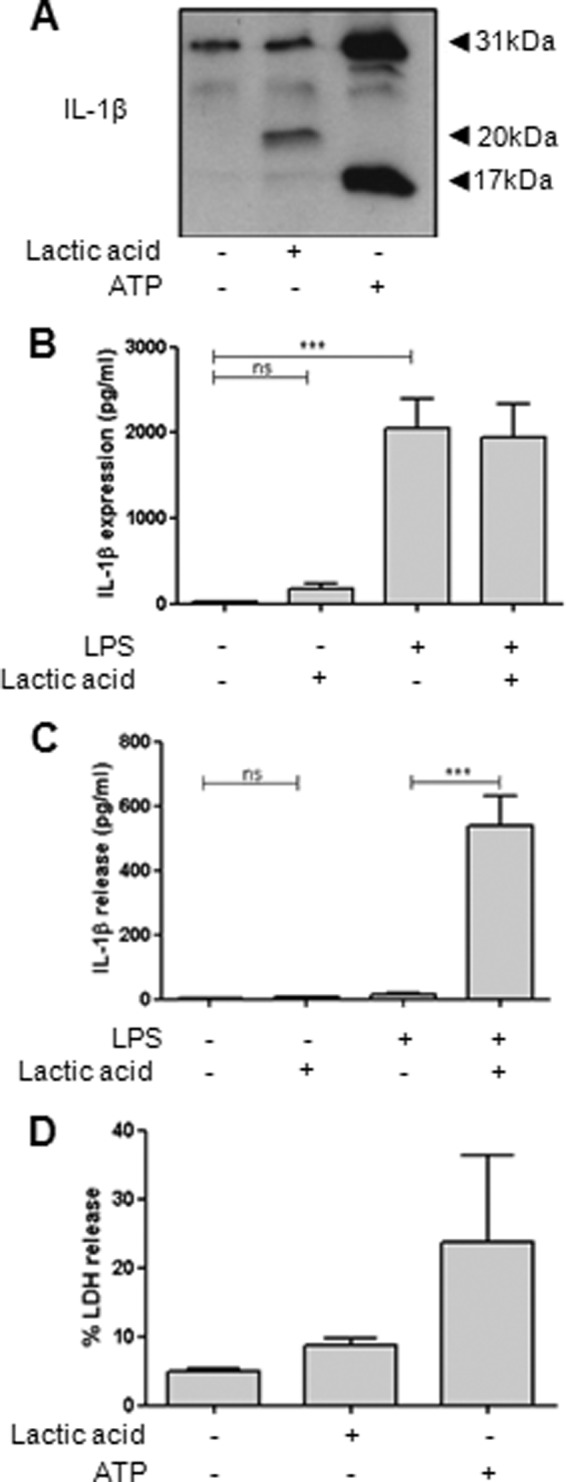
**Lactic acid induces the release of 20-kDa IL-1β from primary mixed glia.** LPS-primed mixed glia were treated with lactic acid (25 mm, 8 h) or ATP (5.5 mm, 1 h). Processing of IL-1β was observed by Western blotting of the supernatants (*A*), with quantification of levels of IL-1β in cell lysates (*B*) and released into the supernatants (*C*) measured by ELISA. The effect of lactic acid and ATP on cell death was measured by the release of LDH (*D*). ELISA data are means ± S.E. of six separate experiments. Western blots are representative of three separate experiments. LDH data are means ± S.E. of three separate experiments. Statistical analysis was performed using a one-way ANOVA with a Bonferroni post hoc test. *ns*, not significant; ***, *p* < 0.001.

During cerebral ischemia, there is a marked elevation in lactic acid that contributes to the drop in tissue pH (12, 19). Thus, we hypothesized that lactic acid could stimulate the release of IL-1β in an ischemic brain. Treatment of LPS-primed mixed glia with lactic acid induced the release of 20-kDa IL-1β and a minor amount of 17-kDa IL-1β (Fig. 6*A*). This effect required LPS priming because lactic acid failed to induce expression or release of IL-1β in its absence (Fig. 6, *B* and *C*). Again, there was no obvious change in toxicity with lactic acid treatment (Fig. 6*D*), suggesting that the IL-1β release observed with lactic acid cannot simply be explained by toxicity.

**FIGURE 7.**
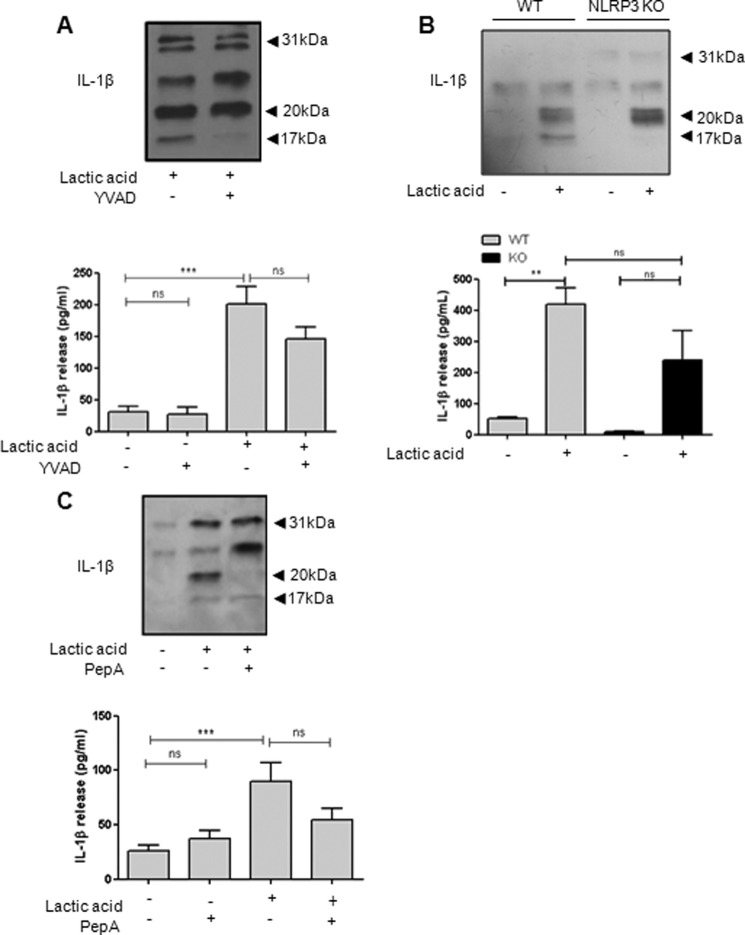
**Lactic acid-induced release of 20-kDa IL-1β occurs independently of caspase-1 in primary mixed glia.** LPS-primed mixed glia were pretreated with 100 μm Ac-YVAD-CHO (*YVAD*; *A*) or 50 μm pepstatin A (*PepA*; *C*) for 15 min prior to lactic acid treatment (25 mm, 8 h). Mixed glial cultures prepared from WT and NLRP3 KO mice were treated with 25 mm lactic acid (8 h; *B*). Processing of IL-1β was observed by Western blotting of the supernatants (*blots*), with quantification of levels released by ELISA (*graphs*). ELISA data are means ± S.E. of between three and six separate experiments. Western blots are representative of three separate experiments. Statistical analysis was performed using a one-way ANOVA with a Bonferroni post hoc test. *ns*, not significant; **, *p* < 0.01; ***, *p* < 0.001.

Lactic acid-induced release of 17-kDa IL-1β was inhibited by Ac-YVAD-CHO, whereas release of 20-kDa IL-1β was unaffected (Fig. 7*A*). Lactic acid also induced the release of 20-kDa IL-1β from NLRP3 KO mixed glia (Fig. 7*B*), confirming the NLRP3 inflammasome- and caspase-1-independent release. Consistent with data presented above, lactic acid-induced 20-kDa IL-1β was inhibited by the cathepsin D inhibitor pepstatin A (Fig. 7*C*).

##### Lactic Acid Induces IL-1α Processing and Release from Mixed Glial Cultures

**FIGURE 8.**
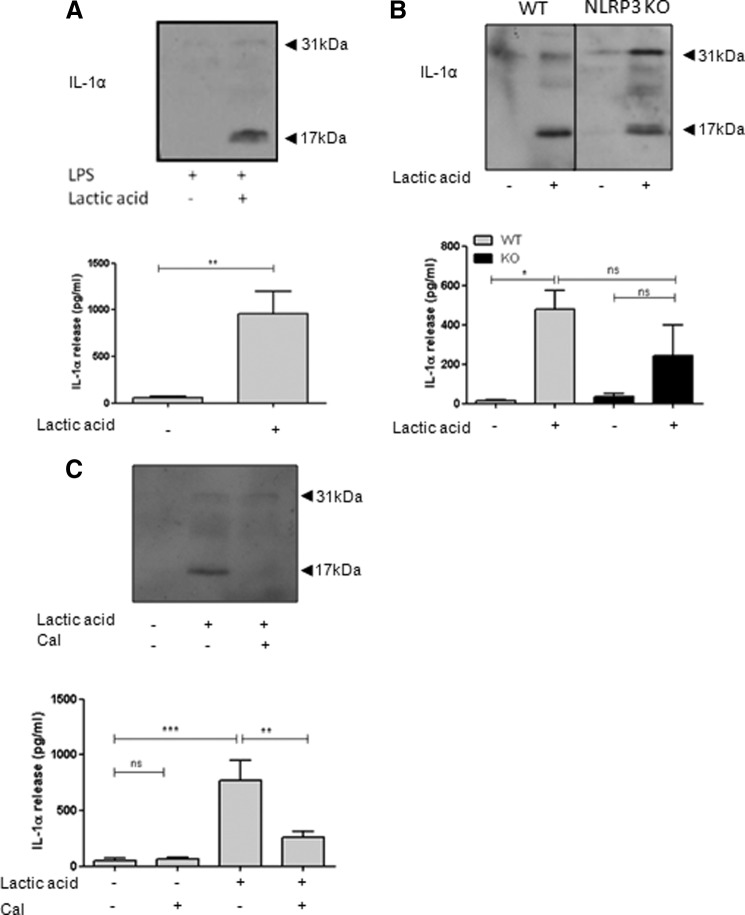
**Lactic acid induces release of IL-1α from primary mixed glia.** LPS-primed WT mixed glia were treated with 25 mm lactic acid for 8 h, and IL-1α processing and release was measured (*A*). LPS-primed mixed glia from WT and NLRP3 KO mice were treated with lactic acid (25 mm; 8 h; *B*). LPS-primed WT mixed glia were pretreated with calpain inhibitor III (*Cal*; 50 μm) for 15 min prior to lactic acid treatment (25 mm, 8 h; *C*). Processing of IL-1α was observed by Western blotting of the supernatants (*blots*), with quantification of levels released by ELISA (*graphs*). ELISA data are means ± S.E. of between three and six separate experiments. Western blots are representative of three separate experiments. Statistical analysis was performed using Student's *t* test (*A*) or one-way ANOVA with a Bonferroni post hoc test (*B* and *C*). *ns*, not significant; *, *p* < 0.05; **, *p* < 0.01; ***, *p* < 0.001.

The effects of lactic acid on the IL-1 system were not limited to IL-1β because treatment of LPS-primed mixed glia with lactic acid also induced the release of processed IL-1α (Fig. 8*A*), which we have reported previously to be a key early mediator of inflammation following acute brain injury (20, 21). Lactic acid-induced IL-1α release was also independent of the NLRP3 inflammasome (Fig. 8*B*) but required the calcium-dependent protease calpain because calpain inhibitor III inhibited lactic acid-induced IL-1α processing and release (Fig. 8*C*). Thus, in line with the NLRP3-independent actions of lactic acid on IL-1β release described above, these data suggest that lactic acid also induces the release of NLRP3-independent but calpain-dependent IL-1α release from glia.

## DISCUSSION

Most studies on IL-1β release in *in vitro* systems are conducted under physiological conditions and in peripheral immune cells such as macrophages. The objective of this study was to investigate IL-1β secretion under disease relevant conditions, with particular reference to ischemic stroke, for which lactic acidosis is relevant (19). Under acidic conditions, “classical” NLRP3 inflammasome-activating DAMPs such as ATP (22) and MSU and CPPD crystals (23) induced the release of a processed IL-1β that was completely independent of the NLRP3 inflammasome and caspase-1, and this occurred in both peripheral macrophage-like cells and also glial cells from the central nervous system. Although we and others have reported that MSU and CPPD stimulate IL-1-independent inflammatory responses (18, 24, 25), when used to stimulate IL-1 production, they are associated with the NLRP3 inflammasome- and caspase-1-dependent processing (23). We also showed that lactic acid induced the release of processed IL-1β that was again independent of the NLRP3 inflammasome and caspase-1. Caspase-1-independent processing of IL-1β has been reported previously and may impact certain diseases even more significantly than caspase-1-processed IL-1β (14–16).

In our experiments, priming to induce the synthesis of pro-IL-1β was required prior to lactic acid (or DAMP)-induced IL-1β release. The TLR4 agonist LPS is a tool for priming cells in culture, but little is known about priming *in vivo* in an injured brain. Brain injury often results in disruption of the blood-brain barrier (6), which would allow circulating peripheral molecules to enter the brain and act on glia. The acute phase protein serum amyloid A is elevated in plasma during inflammation and has been shown to prime glial cells to release IL-1 following subsequent DAMP stimulation *in vitro* in a way similar to LPS (18).

The contribution of IL-1 to ischemic brain injury is well established (6), with the utility of the IL-1 receptor antagonist as a therapeutic under clinical investigation (26). Brain acidosis after ischemia contributes to neuronal injury, and the damaging effects are mediated, at least in part, by acid-sensing ion channels (27, 28). Our data provide the rationale that acidosis and neuronal injury could also be linked via the IL-1 system. Levels of lactic acid increase in an ischemic brain due to a lack of oxygen and the up-regulation of anaerobic glycolysis. Stimulation of LPS-primed cultures of glial cells with lactic acid or with NLRP3 inflammasome-activating DAMPs under acidic conditions induced cathepsin D-dependent release of 20-kDa IL-1β, consistent with a previous report showing ATP-induced release of 20-kDa IL-1β from microglia under acidic conditions (13). This cathepsin D cleavage of IL-1β adds to a growing number of proteases now known to cleave pro-IL-1β. These proteases cleave IL-1β at different sites to produce different size fragments corresponding to differing activities (29). The putative cathepsin D-dependent product at 20 kDa is suggested to be ∼5-fold less active at IL-1 receptor I than the caspase-1-generated 17-kDa product (29). Thus, if generated *in vivo*, it could act as an agonist or maybe a partial agonist of IL-1 receptor I and contribute to inflammatory responses during ischemia; therefore, further experiments to understand the exact nature of its role *in vivo* are required to help direct any future targeting of this pathway. Acidosis was also recently described as an inducer of the NLRP3 inflammasome (17). Although we did observe 17-kDa IL-1β following lactic acid treatment (*e.g.* Fig. 7*A*), it was the minor species compared with the 20-kDa form. Together, these data strongly suggest that acidosis is an important disease relevant regulator of IL-1β-processing pathways.

Until recently, the precursor of IL-1α (pro-IL-1α) was thought to be as biologically active as the mature 17-kDa form at IL-1 receptor I. However, recent research has shown that processed IL-1α is significantly more biologically active than the pro-form (30, 31), which highlights IL-1α processing as a biologically important mechanism. In addition to its effects on IL-1β, lactic acid also induced the release of processed IL-1α, which was 17 kDa and was inhibited by a calpain inhibitor, consistent with the involvement of a calpain protease in its processing (7). DAMPs are known to induce the secretion of processed IL-1α (32), and DAMP-induced processing and secretion of IL-1α can be dependent, partially dependent, or independent of the NLRP3 inflammasome and caspase-1, depending upon the DAMP (32). Our data suggest that lactic acid-induced IL-1α secretion occurs independently of the inflammasome. In addition to the effects of IL-1β in acute brain injury, IL-1α is also known to play an important role in brain injury (20, 21, 33). IL-1α is up-regulated before IL-1β and as early as 4 h post-ischemic brain injury in areas of subsequent neuronal loss, suggesting that it may be the key driver of the damaging early inflammatory response (20). Thus, the lactic acid-induced IL-1α release we have described may contribute to damage post-ischemic injury.

In summary, these data provide new insights into the mechanisms of IL-1 production during disease relevant conditions. We have shown that under acidic conditions, both proinflammatory forms of the IL-1 family (IL-1β and IL-1α) are regulated independently of the NLRP3 inflammasome. Thus, consideration of this observation should be made when investigating inhibitors of the NLRP3 inflammasome to target IL-1 in disease, particularly where changes in the local environment may dictate the mechanism of IL-1β processing.

## Footnotes

2 The abbreviations used are:

DAMP: damage-associated molecular pattern
Ac-YVAD-CHO: acetyl-YVAD-aldehyde
MSU: monosodium urate
CPPD: calcium pyrophosphate dihydrate
KO: knock-out
LDH: lactate dehydrogenase
ANOVA: analysis of variance.
